# Enhanced function and quality of life following 5 months of exercise therapy for patients with irreparable rotator cuff tears – an intervention study

**DOI:** 10.1186/s12891-016-1116-6

**Published:** 2016-06-08

**Authors:** Birgitte Hede Christensen, Kathrine Skov Andersen, Sten Rasmussen, Elizabeth Lykholt Andreasen, Lotte Mejlvig Nielsen, Steen Lund Jensen

**Affiliations:** Department of Physio and Occupational Therapy, Aalborg University Hospital, Aalborg, Denmark; Orthopaedic Surgery Research Unit, Aalborg University Hospital, Aalborg, Denmark; Department of Neurology, Region Hospital Viborg, Viborg, Denmark; Department of Clinical Medicine, Aalborg University, Aalborg, Denmark; Department of Orthopaedic Surgery, Aalborg University Hospital, Aalborg, Denmark

**Keywords:** Rotator cuff rupture, Conservative management, Rehabilitation, Exercise therapy, Tendon injuries

## Abstract

**Background:**

Rotator cuff rupture is associated with dysfunction, pain and muscular weakness related to the upper extremity. Some evidence exists to support the beneficial effect of exercises but there is lack of evidence of which exercises imply the best effect and how physiotherapy should be administered. Therefore, the purpose of this study was to examine the effect of a neuromuscular exercise program for patients with irreparable rotator cuff rupture.

**Methods:**

Based on sample-size calculations thirty patients with chronic irreparable rotator cuff tears (of at least m. supraspinatus and m. infraspinatus) was consecutively included. Twenty-four patients completed the five months training to restore function with focus on centering the humeral head in the glenoid cavity trough strengthening m. deltoideus anterior and m. teres minor. The primary outcome measure was Oxford Shoulder Score which was completed at baseline, 3 and 5 months follow-up. One-way, repeated-measure ANOVA was used if data was normally distributed. Secondary outcome measures included EQ-5D, range of motion, strength and muscle activity. Paired t-test and Wilcoxon Signed Ranks Test was used to the appropriate outcomes.

**Results:**

Improvements was seen for both primary and secondary outcomes from baseline to follow-up. Oxford Shoulder Score improved from 25.6 (SD 8.1) at baseline to 33.8 (SD 8.7) at 3 months (*p* = 0.004) and 37.2 (SD 8.2) at five months (*p* < 0.001). Range of motion in abduction significantly increased by 34.4° (95 % CI: 11.6–57.2). Strength measured in flexion 45, flexion 90 and abduction also significantly increased at 5 months by 10.2 (95 % CI: 0.8–19.6), 7.0 (95 % CI: 0.0–14.0) and 12.3 (95 % CI: 3.4–21.3) respectively. The remaining outcomes for range of motion and strengths only showed small and non-significant changes. Furthermore patients reported higher levels of quality of life and reduced level of pain after five months.

**Conclusion:**

Following a five months exercise protocol patients with irreparable rotator cuff tears showed increased function in their symptomatic shoulder, reduced pain and increased quality of life. This study therefore supports the use of exercise therapy in patients with irreparable rotator cuff rupture.

**Trial registration:**

This study is approved by The National Committee on Health Research Ethics (N-20120040) and registered retrospectively at ClinicalTrials.gov in April 2016 (NCT02740946).

**Electronic supplementary material:**

The online version of this article (doi:10.1186/s12891-016-1116-6) contains supplementary material, which is available to authorized users.

## Background

Irreparable rotator cuff rupture is associated with symptoms such as dysfunction, chronic pain and muscular weakness [1]. Literature indicates that the prevalence of rotator cuff ruptures are increasing with increasing age [2], but there is a lack of evidence for the most efficient treatment [1]. Due to the degenerative nature of the lesion and muscle atrophy with tendon retraction, tendon repair may not be possible. Ainsworth and Lewis [3] also concluded in a systematic review (2007), that only some evidence exists to support the use of exercises in treating patient with full thickness rotator cuff tears, but there is a lack of evidence of which exercises imply the best effect and how physiotherapy should be administered [3, 4].

Observational studies indicate that increased strength of the anterior part of m. deltoideus has a positive effect on symptoms in these patients [3, 5] and that achievement of active lateral rotation also imply better outcome [6]. Furthermore, Baydar et al. investigated the effect of a substantial exercise program for twenty patients with full-thickness rotator cuff tears, consisting of three phases with passive exercises, then strengthening exercises and finally return to normal activities. The results showed significant improvement with regards to range of motion, pain and function after six months [7]. On the other hand Collin et al. investigated the effect of a rehabilitation program for patients with irreparable massive rotator cuff tears in a prospective intervention study, but found no indication for strengthening m. deltoideus anterior [8]. Instead they suggest strengthening of the scapula stabilizers and the entire m. deltoideus [8]. In agreement with the literature Collin et al. also indicate that there are no consensus regarding non-operative treatment modalities such as methods, duration and indications [8].

Based on the observations that m. deltouideus anterior and m. teres minor are of particular importance in compensating for rotator cuff tears [6], we hypothesized that a rehabilitation program including strengthening exercises for these muscles would be beneficial. The literature also indicates that an undesired increased neuromuscular activity of m. trapezius superior often is seen in patients with shoulder dysfunction [9–11]. We therefore hypothesized that the neuromuscular activity of m. trapezius superior would far exceed the activity from m. deltoideus anterior at baseline based on compensatory strategies during movement of the symptomatic upper extremity and that this imbalance would equalize as the strength of m. deltoideus anterior would improve.

Hence, the aim of this study was to examine the effect of a neuromuscular exercise program including strengthening exercises of m. deltouideus anterior and m. teres minor on strength, range of motion, pain, self-reported shoulder function and quality of life in patients with irreparable rotator cuff tear. Secondly, we aimed to measure the effect such a program would have on neuromuscular activity.

## Methods

A consecutive inclusion of patients started in August 2011 and the last patient ended the training in December 2014. Patients were included if they had experienced symptoms of rotator cuff rupture for at least three months with rupture of minimum m. supraspinatus and m. infraspinatus visualized by ultrasonography or arthroscopy, no neurological conditions which could affect muscle strength or activity and were able to read and understand Danish. Both patients with and without a history of shoulder trauma were included. The rotator cuff tears were classified as irreparable by an experienced shoulder surgeon based on clinical evaluation (muscle atrophy) and ultrasonography. If no tendon tissue was visible on ultrasonography, the rotator cuff tear was regarded as irreparable. If in doubt, patients underwent MRI or arthroscopy. Muscle atrophy with fatty infiltration together with severe tendon retraction on MRI were indicative for irreparability. During arthroscopy, if the tendons were poor quality or if they after release could not be pulled to their insertion, they were regarded as irreparable. Patients were excluded if former rotator cuff surgery had been performed or if they had a history of shoulder fracture or inflammatory conditions in the shoulder. Patients were also excluded if they had significant osteoarthritic changes on antero-posterior and/or lateral (outlet view) radiographs.

Patients were recruited from the Shoulder and Elbow Clinic, Aalborg University Hospital by an orthopedic surgeon based on the inclusion criteria. The study was performed in accordance with the Declaration of Helsinki and approved by the Danish Data Protection and The National Committee on Health Research Ethics (N-20120040). Furthermore an informed consent was obtained from all patients prior to participation and the rights of the patients were protected.

### Sample size

Sample size calculations were based on a minimal clinical important change of 5 point and a standard deviation of 6.2 point on the Oxford Shoulder Score [12, 13]. Patients included act as their own controls whereas a paired design is applicable. However, sample size calculation was based on an unpaired design to increase strength. The alpha level was set at 5 % and the beta level set at 80 %. With these values calculations showed that a minimum of 24 patients were needed. To account for possible drop-outs 30 patients were included consecutively.

### Rehabilitation protocol

All patients received patient education by a physiotherapist including information on the diagnosis and rationale for the exercise protocol. Furthermore patients were informed on how to manage pain related to the exercises; i.e. any increased pain experienced during exercises must drop to pre-exercise level after 30 min of rest, otherwise exercises was adjusted to a less challenging level.

Patients were instructed to perform two different exercises three times a week in a total of five months. One of the training sessions each week was supervised by a physiotherapist for the first three months and one session every second week was supervised for the last two months. To strengthen compliance all patients filled out a training log after each training session. This included reporting of pain on a visual analogue scale (VAS) before, during and after performing the exercises. Use of pain medicine was also registered by the patients.

The exercise program consisted of one exercise for m. deltoideus anterior and one for m. teres minor including 2–3 min of warm-up before starting the exercise program.

**Fig. 1:**
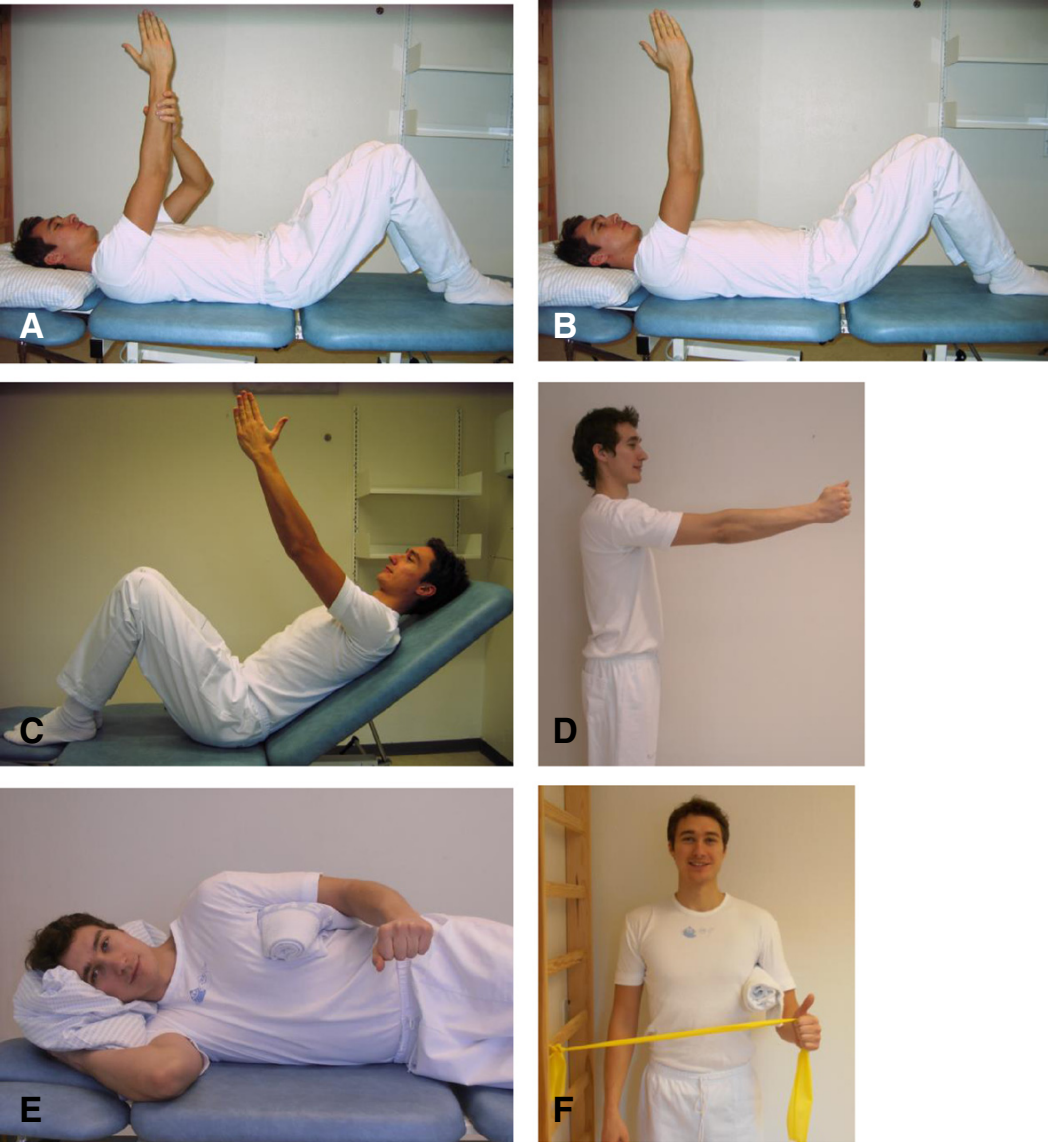
Exercises protocol. **a**-**d** shows the exercises for m. deltoideus anterior in different stages. First with external support (**a**), then changes in the starting position and thereby the load and gravity of the exercise (**c**-**d**) and finally external weight could be applied. From the positions illustrated the patients performed flexion. **e**-**f** shows the exercises for m. teres minor again challenging the patient by changing the starting position and adding external load. From the positions illustrated the patient performed external rotation. Consent to the use of the images has been obtained from the subject

Each week the physiotherapist graduated the exercises to the patient’s ability according to pain during and after the exercise and whether or not the patient felt challenged during the exercises. The physiotherapist could stepwise increase exercise load by removing external support, by changing the starting position and thereby the gravitational load of the shoulder, by increasing the number of repetition (to a maximum of 4x12 before next level was applied) or by adding external load to the exercise (including gravity) (see exercise protocol in Fig. 1). During the performance of the exercises the physiotherapist instructed patients to center the humeral head in glenoid cavity, stabilize scapula and perform the exercises in a slow and controlled pace.

### Outcome measures

The primary outcome measure was the Oxford Shoulder Score which is a self-reported questionnaire containing 12 questions regarding level of function. Oxford Shoulder Score is translated to Danish, and the translated edition is both valid and reliable [14]. Patients reported their level of function on the Oxford Shoulder Score at baseline, 3 and 5 months follow-up.

The secondary outcome measures included the EQ-5D-5L questionnaire, active range of motion, strength measured with a hand-held dynamometer and muscle activity measured with surface electromyography (EMG).

The EQ-5D-5L is a standardised questionnaire to measure health status. It includes questions on mobility, self-care, usual activities, pain/discomfort and anxiety/depression as well as an overall measure of health related quality of life rated on a scale form 0–100 with higher values indicating higher health status [15].

Range of motion was measured in flexion, abduction and external rotation using a goniometer. Furthermore, a dynamic flexion was conducted where the subjects were asked to perform maximum isolated shoulder flexion with no compensatory movement of the torso. A marker was set at the wall next to the patients hand when they reached maximum flexion and the height from the floor to the marker was registered. After each performance patients were to report their pain on VAS.

Strength was measured in flexion (45 and 90°), abduction, internal and external rotation of the shoulder. A hand-held dynamometer (PowerTrack II, JTech Medical Industries, Salt Lake City, USA) was calibrated and used. The dynamometer started the registration when 4.4 Newton was reached. After each performance patients were to report their pain on VAS.

Muscle activity was measured in m. trapezius superior and m. deltoideus anterior with surface EMG. The muscle activity of these muscles was recorded during flexion and abduction at 45° and flexion at 90°. The procedure for electrode placement is described in a previous publication [16]. The EMG signals were amplified with a gain of 2000 and filtered (Biovision EMG equipment, Werheim, Germany) using a [10–400 Hz] band-pass filter (4^th^ order Butterworth filter). The input voltage was ± 10 V. Then, the EMG was digitally sampled at 2000 Hz with a 12 bit AD converter (Biovision, Werheim, Germany). The software program DASYLab 10.0 was used for data collection.

The secondary outcomes measures were performed at baseline and 5 months follow-up. The same physiotherapists performed the baseline and follow-up measurements and were blinded to the results from baseline when performing the follow-up measurement.

Range of motion was measured first followed by measurements of strength and the dynamic flexion. In which order the specific movement directions was performed during e.g. range of motion and strength was randomized by closed opaque envelopes. For further information on the test positions used in this study and the reliability on the hand-held dynamometer and EMG see Andersen et al. [16].

### Statistical analysis

Normal distribution was visually inspected using QQ-plots.

Data from the Oxford Shoulder Score was transformed according to guidelines [17, 18] and if normally distributed a one-way, repeated-measure ANOVA was used, with Bonferroni as post-hoc test. When transformed, score can range from 0–48, with higher values indicating better outcome.

Results from EQ-5D-5L is presented as index values and VAS as a measure of overall self-rated health status [19]. The statistical analysis was performed with Wilcoxon Signed Ranks Test.

Data from the goniometer and hand-held dynamometer were imported to Excel 2003 (Microsoft Office©). A mean of the last three of four repetitions for the hand-held dynamometer and the dynamic flexion was used in the statistical analysis. If data from the goniometer, the hand-held dynamometer and the dynamic flexion were normally distributed a paired t-test was used. If the data was not normally distributed a Wilcoxon Signed Ranks Test was used. As describes in the literature pain reported on VAS was considered ordinal and therefore a Wilcoxon Signed Ranks Test was used [20].

Data from the EMG were analyzed in Matlab 2011a (MathWorks, Massachusetts, U.S.A.), using a costume made script. The data were digitally filtered ([10–300] Hz, 4th order Butterworth and Notch Filter with a width of 1 Hz at a frequency of 50 Hz). A mean of the last three contractions was used in the statistical analysis and normalized to a maximum isometric contraction. For further information on the normalization procedure see Andersen et al. [16].

The statistical analysis was performed in SPSS 22 and a *p*-value <0.05 was considered significant.

## Results

The study included 30 patients with rotator cuff tears, 10 women and 20 men, mean age 70,4 years (ranged 49–89), mean BMI 28 (ranged 20.8–35.8). The left shoulder was the symptomatic shoulder for six patients and the average symptom duration was 38.6 months (ranged 3–216 months). 53 % of the patient reported to have had symptoms for more than one year.

The diagnosis of the tear were in all cases made by ultrasonography. In addition, four patients underwent MRI and seven patients arthroscopy for verification of the tear and its irreparability. All patients had complete rupture of the supraspinatus tendon, and all patients had a rupture of the infraspinatus tendon, either completely (27 patients) or partial (3 patients). Six patients had an additional tear of the subscapularis tendon.

Twenty-four patients had no radiographic signs osteoarthritis, five patients had as the only finding an osteophyte of maximum 2 mm at the humeral neck, and one patient had a small osteophyte at the humeral neck and reduced joint width inferiorly.

Six patients failed to complete the intervention and the results are therefore based on 24 patients. Reasons for failure to complete the treatment were for two patients problems with getting to the training location, one patient was referred again to the orthopedic department for operation and for three patients the reason was unknown.

**Fig. 2:**
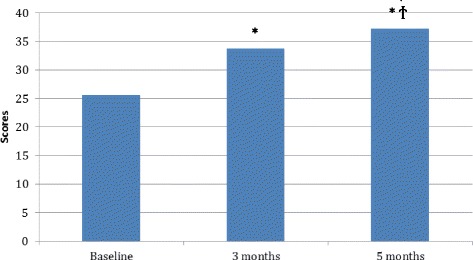
Scores on the Oxford Shoulder Score from baseline, 3 and 5 months. Data are presented as mean and standard deviations. * significant difference from baseline. ϯ significant difference from 3 months

Oxford Shoulder Scores showed significant improvements from baseline to 3 months follow-up with a mean increase of 8.2 (95 % CI: 2.4–14.0), from baseline to 5 months follow-up with a mean increase of 11.7 (95 % CI: 8.7–16.6) and from 3 months to 5 months follow-up with a mean increase of 3.4 (95 % CI: 0.9–5.9) (see Fig. 2) (Additional file 1: Table S1).

The secondary outcomes also showed improvements. EQ-5D index values increased significantly from a median of 0.671 at baseline to 0.755 at 5 months follow-up (*p* = 0.009). EQ-5D VAS also increased with a median of 60.0 at baseline to 80.0 at 5 months follow-up (*p* < 0.001) (Additional file 2: Table S2).

Range of motion increased significantly for abduction whereas the increases were small and non-significant for flexion and external rotation (see Table 1). On the other hand pain reported on VAS during abduction, flexion and external rotation all decreased significantly from baseline to 5 months follow-up (see Table 1) (Additional file 3: Table S3).

**Table 1:** Range of motion

	Baseline mean (SD)	5 months mean (SD)	Difference mean (95 % CI)	Difference *p*-value
Abduction	93.7 (38.9)	128.1 (52.7)	34.4 (11.6–57.2)	0.005*
Flexion	132.5 (8.0)	133.9 (60.0)	1.4 (−24.1–26.8)	0.912
External rotation	27.9 (18.6)	31.7 (15.7)	3.8 (−4.7–12.4)	0.364
	Baseline median (25th ; 75th percentiles)		5 months median (25th, 75th percentiles)	Difference p-value
VAS abduction	5.0 (3.0;6.3)		2.0 (1.0;4.0)	0.001*
VAS flexion	5.0 (3.8;7.3)		1.0 (0.0;3.0)	<0.001*
VAS external rotation	4.0 (1.8;6.3)		0.5 (0.0;3.8)	0.015*

Range of motion presented as mean and standard deviations (SD) and pain reported on a visual analogue scale (VAS) presented as median, 25^th^ and 75^th^ percentiles during the same movement directions. The outcome measures were performed at baseline and 5 months follow-up. *CI* Confidence interval. * indicates significant difference

Strength measured with the hand-held dynamometer showed significant increases for abduction and flexion at 45 and 90°. Strength in internal and external rotation also increased but only limited and non-significant (see Table 2).

**Table 2:** Strength measured on a hand-held dynamometer and pain measured on a visual analogue scale

	Baseline mean (SD)	5 months mean (SD)	Difference mean (95 % CI)	Difference *p*-value
Abduction	50.2 (32.5)	62.5 (34.1)	12.3 (3.4–21.3)	0.009*
Flexion 45°	23.5 (15.9)	33.7 (22.8)	10.2 (0.8–19.6)	0.036*
Flexion 90°	17.6 (8.4)	24.6 (17.0)	7.0 (0.0–14.0)	0.049*
Internal rotation	93.1 (47.4)	102.1 (54.6)	9.0 (−1.9–19.8)	0.102
	Baseline median (25th; 75th percentiles)	5 months median (25th; 75th percentiles)		Difference *p*-value
External rotation	24.2 (7.0;44.7)	24.9 (11.0;44.7)		0.363
	Baseline median (25th; 75th percentiles)	5 months median (25th; 75th percentiles)		Difference *p*-value
VAS abduktion	5.3 (3.0;6.5)	4.3 (3.0;7.0)		0.655
VAS flexion 45°	6.5 (4.4;8.0)	2.7 (1.0;5.0)		<0.001*
VAS flekxion 90°	5.3 (4.2;8.2)	3.0 (0.5;5.0)		0.001*
VAS internal rotation	3.0 (0.0;4.3)	0.2 (0.0;2.8)		0.047*
VAS external rotation	4.5 (2.0;7.0)	1.0 (0.0;4.3)		0.001*

Strength measured in Newton presented as mean and standard deviations (SD) for normally distributed values. If not normally distributed data is presented as median, 25^th^ and 75^th^ percentiles (external rotation). Pain reported on a visual analogue scale presented as median and 25^th^ and 75^th^ percentiles. *CI* Confidence interval. * indicates significant difference

Flexion at 90° was only performed for 13 patients since the movement caused too much pain in the rest of the subjects for them to carry on. Pain reported on VAS significantly decreased during all movement directions except from abduction (see Table 2) (Additional file 4: Table S4).

Height (cm) of the dynamic flexion was not normally distributed. Median height in centimeters at baseline was 167.2 and increased significantly to 199.5 at 5 months follow-up (*p* < 0.001) (Additional file 5: Table S5).

The results from the EMG data only showed a significant difference for flexion 90° of m. deltoideus anterior, where the activity presented as normalized values showed decreased activity from baseline to 5 months follow-up (see Table 3). For the rest of the movement directions no significant or clinical relevant differences were seen (Additional file 6: Table S6).

**Table 3:** Electromyography during isometric abduction, isometric and dynamic flexion

	m. trapezius - mean (SD)		m. deltoideus – mean (SD)	
	Baseline	5 months	Baseline	5 months
Abduction	138.1 (182.2)	147.2 (179.1)	123.8 (60.2)	141.0 (88.8)
Flexion 45°	108.4 (127.2)	313.6 (909.2)	134.8 (81.2)	117.0 (83.0)
Flexion 90°	169.9 (213.9)	179.4 (158.3)	138.1 (32.7)	109.8 (27.2)*
Dynamic flexion	130.2 (184.2)	147.4 (163.3)	108.1 (52.8)	110.5 (54.8)

Data from the electromyography of m. trapezius superior and m. deltoideus anterior. Data are normalized to a maximum isometric contraction and therefore displayed as present of a reference value. Data are presented as mean and standard deviations (SD). Only 14 patients were able to perform flexion 90°. * indicates significant difference (here *p* = 0.021)

## Discussion

Our results show that patients with irreparable rotator cuff rupture significantly improve their self-reported shoulder function and quality of life following five months of exercise therapy. Also the objective outcome measures showed significant improvements regarding strength and pain, however changes in range of motion was limited and no relevant changes was seen for the muscle activity measured with surface EMG. The dynamic flexion reached significant increase from baseline to follow-up with a median increase of 32.3 cm, which potentially can have a considerable effect of the patients’ abilities during daily activities.

Patients included in this study had long lasting symptoms with a mean duration of 38.6 months. Fourteen patients had consulted with a physiotherapist before entering the study but only eight of these reported that they had tried exercise therapy and with only limited or transient effect. The rest of the patient who had tried physiotherapy reported that massage, stretching, manual mobilization and acupuncture was among the chosen treatment strategies and again with only limited effect. This lack of consistency in whether or not treatment was applied and if, how it should be administered matches the lack of consensus regarding non-operative treatment modalities found in the literature [3, 4, 8, 21].

Only a limited number of studies exist in the literature regarding exercise therapy for this patient group. Collin et al. reported significant improvement on the Constant score at two years follow-up for patient with massive rotator cuff rupture [8]. Furthermore they found that patients with posterior tears (m. supraspinatus and m. infraspinatus) were the ones who benefitted the most from the exercise therapy [8]. In contrary to our hypothesis Collin et al. does not support the strengthening of the anterior part of the deltoid muscle but prefer to strengthen the entire deltoid muscle as well as the muscles that stabilize the scapula. In line with the results from our study both Ainsworth [6] and Levy et al. [5] found significant improvement on the Oxford Shoulder Disability Questionnaire and the Constant Score respectively following exercise therapy focusing on the anterior part of the deltoid muscle indicating the importance of this muscle in compensating for the reduced function of the rotator cuff. Also Baydar et al. found positive results from exercise therapy for patients with full-thickness tears indicating the overall beneficial effects of this conservative management [7]. However the description of the exercise program used in the study by Baydar et al. is inadequate and therefore a more detailed comparison is not possible. Furthermore, in line with our study Baydar et al. lacks a control group, which also weakens the results [7].

To the best of our knowledge this study is the first to record muscle activity using surface EMG following an exercise program for patients with rotator cuff tears. The results from the EMG data cannot support our hypothesis that the activity of m. trapezius superior would decrease and that the activity of m. deltoideus anterior would increase following the exercise protocol. However the wide standard deviations reflect the large variability of these data and therefore no conclusions can be made. The activity from the EMG increases from baseline to 5 months follow-up, especially with regard to m. trapezius superior, indicating to increase function of the shoulder in these patients a general increase in muscle activity surrounding the shoulder is needed.

The strength of our study is that the exercise protocol is simple and contains only two exercises and one warm-up exercise. A large effort was made in standardizing the exercises and ensuring that all patients received the same treatment. Some of the data are not normally distributed and large standard deviations were also seen indicating that the sample size was too small or that the accuracy of outcome measures used was not sufficient.

To our knowledge no randomized controlled trial has been conducted, but the majority of the existing intervention studies support the use of exercise therapy for treatment in these patients.

A potential weakness of this study is also that there was no control group, and we do not know the natural course of the symptoms in the patient group. Spontaneous improvement, however, seems less likely since the patients have had symptoms for a long period (more than 3 years on average). We therefore find it likely that the observed improvement was due to a beneficial effect of the exercise program, and recommend that it is tried in patients with irreparable rotator cuff lesions.

## Conclusion

Five months of exercise therapy focusing on strengthening m. deltoideus anterior and m. teres minor for patient with irreparable rotator cuff rupture resulted in improved patient-reported function and quality of life even after long symptom duration. Furthermore, increased strength and reduced pain was measured at five months. Our results indicate that exercise therapy is beneficial and should be offered to patients with rotator cuff rupture when considering non-operative management. Randomized controlled trials are warranted before final conclusions can be made.

## Abbreviations

EMG, electromyography; VAS, visual analogue scale

## Additional files

Additional file 1: Table S1. Oxford Shoulder Score. (XLSX 12 kb) Additional file 2: Table S2. EQ-5D-5L. (XLSX 10 kb) Additional file 3: Table S3. Range of motion. (XLSX 15 kb) Additional file 4: Table S4. Strength. (XLSX 20 kb) Additional file 5: Table S5. Dynamic flexion. (XLSX 10 kb) Additional file 6: Table S6. EMG. (XLS 134 kb)

## References

[1] Downie B, Miller B (2012). Treatment of rotator cuff tears in older individuals: a systematic review. J Shoulder Elb Surg.

[2] Pegreffi F, Paladini P, Campi F, Porcellini G (2011). Conservative management of rotator cuff tear. Sport Med Arthrose Rev.

[3] Ainsworth R, Lewis JS (2007). Exercise therapy for the conservative management of full thickness tears of the rotator cuff: a systematic review. Br J Sports Med.

[4] Lin J, Weintraub N, Aragaki D (2008). Nonsurgical treatment for rotator cuff injury in the elderly. Am Med Dir Assoc.

[5] Levy O, Mullett H, Roberts S, Copeland S (2008). The role of anterior deltoid reeducation in patients with massive irreparable degenerative rotator cuff tears. J Shoulder Elb Surg.

[6] Ainsworth R, Srp F, Hons B (2006). Physiotherapy rehabilitation in patients with massive, irreparable rotator cuff tears. Musculoskeletal Care.

[7] Baydar M, Akalin E, El O, Gulbahar S, Bircan C, Akqul O (2009). The efficacy of conservative treatment in patiens with full-thichness rotator cuff tears. Rheumatol Int.

[8] Collin P, Gain S, Nguyen Huu F, Lädermann A (2015). Is rehabilitation effective in massive rotator cuff tears?. Orthop Traumatol Surg Res.

[9] Cools A, Dewitte V, Lanszeert F, Notebaert D, Roets A, Soetens B (2007). Rehabilitation of scapular muscle balance: which exercises to prescribe?. Am J Sport Med.

[10] Hawkes D, Alizadehkhaiyat O, Kemp G, Fisher A, Roebuck M, Frostick S (2012). Shoulder muscle activation and coordination in patients with massive rotator cuff tear: An electromyographic study. J Orthop Res.

[11] Ludewig P, Reynolds J (2009). The association of scapular kinematics and glenohumeral joint pathologies. J Orthop Sport Phys Ther.

[12] Christiansen D, Frost P, Falla D, Haahr J, Froch L, Svendsen S (2015). Responsiveness and Minimal Clinically Important Change: A Comparison Between 2 Shoulder Outcome Measures. J Orthop Sport Phys Ther.

[13] Dawson B, Miller B (2002). Comparison of Clinical and Patient-Based Measures to Assess Medium-Term Outcomes Following Shoulder Surgery for Disorders of the Rotator Cuff. Arthritis Rheum.

[14] Frich L, Noergaard P, Brorson S (2011). Validation of the Danish version of Oxford Shoulder Score. Dan Med Bul.

[15] Oemar M, Janssen B (2013). EQ-5D-5L User Guide Basic information on how to use the EQ-5D-5L instrument.

[16] Andersen K, Christensen B, Samani A, Madeleine P (2014). Between-day reliability of a hand-held dynamometer and surface electromuography recordings during isometric submaximal contractions in different shoulder positions. J Electromyogr Kinesiol.

[17] Dawson J, Rogers K, Fitzpatrick R, Carr A (2009). The Oxford shoulder score revisited. Arch Orthop Trauma Surg.

[18] Isis Innovation Limited. Scoring System for the Oxford Shoulder Score. 2011. http://isis-innovation.com/outcome-measures/oxford-shoulder-score-oss/.

[19] van Hout B, Janssen M, Feng Y, Kohlmann T, Busschbach J, Golicki D (2012). Interim scoring for the EQ-5D-5L: Mapping the EQ-5D-5L to EQ-5D-3L value sets. Value Heal.

[20] McCrum-Gardner E (2008). Which is the correct statistical test to use?. Br J Oral Maxillofac Surg.

[21] Green S, Buchbinder R, Hetrick S (2003). Physiotherapy interventions for shoulder pain. Cochrane Database Syst Rev.

